# Do the diverse phenotypes of Prader-Willi syndrome reflect extremes of covariation in typical populations?

**DOI:** 10.3389/fgene.2022.1041943

**Published:** 2022-11-24

**Authors:** Iiro Salminen, Silven Read, Bernard Crespi

**Affiliations:** Crespi Laboratory, Department of Biological Sciences, Simon Fraser University, Vancouver, BC, Canada

**Keywords:** Prader-Willi and Angelman syndromes, hypothalamus, genomic imprinting, schizophrenia, attachment, sleep, feeding

## Abstract

The phenotypes of human imprinted neurogenetic disorders can be hypothesized as extreme alterations of typical human phenotypes. The imprinted neurogenetic disorder Prader-Willi syndrome (PWS) features covarying phenotypes that centrally involve altered social behaviors, attachment, mood, circadian rhythms, and eating habits, that can be traced to altered functioning of the hypothalamus. Here, we conducted analyses to investigate the extent to which the behavioral variation shown in typical human populations for a set of PWAS-associated traits including autism spectrum cognition, schizotypal cognition, mood, eating, and sleeping phenotypes shows covariability that recapitulates the covariation observed in individuals with PWS. To this end, we collected data from 296 typical individuals for this set of phenotypes, and showed, using principal components analysis, evidence of a major axis reflecting key covarying PWS traits. We also reviewed the literature regarding neurogenetic syndromes that overlap in their affected traits with PWS, to determine their prevalence and properties. These findings demonstrate that a notable suite of syndromes shows phenotypic overlap with PWS, implicating a large set of imprinted and non-imprinted genes, some of which interact, in the phenotypes of this disorder. Considered together, these findings link variation in and among neurogenetic disorders with variation in typical populations, especially with regard to pleiotropic effects mediated by the hypothalamus. This work also implicates effects of imprinted gene variation on cognition and behavior in typical human populations.

## Introduction

The hypothalamus is a highly conserved region in vertebrate brains, that has been found to regulate physiological homeostasis *via* several neural mechanisms and specific signaling cascades, including biorhythmicity, sleep-wake control and the regulation of satiety and hunger states [reviewed in Xie and Dorsky (2017) and Arrigoni et al. (2019)]. The primary functions of the hypothalamus have been highlighted by syndromes involving hypothalamic dysfunction, which demonstrate phenotypes involving disrupted metabolic control and altered regulation of sleep (Ivanova and Kelsey, 2011; Angulo et al., 2015). Furthermore, mice model studies that involve knockouts of genes active in the hypothalamus tend to show altered phenotypes of food intake, sleep or both (Kozlov et al., 2007; Ivanova and Kelsey, 2011; Mercer et al., 2013; Qi et al., 2015; Pace et al., 2020). Recent gene knockout studies also appear to show that hypothalamic neuron populations primarily expressing a specific neural transmitter may simultaneously exert pleiotropic effects on several behaviorally distinct aspects of energy homeostasis. For example, model mice with a specific lack of expression for the melatonin-concentrating hormone (MCH), typically expressed exclusively in the hypothalamus, show a pleiotropic phenotype with increased wakefulness and an increase in activity during fasting periods, which may indicate that MCH neurons typically function to promote sleep and suppress food-seeking behaviors for conservation of energy balance (Willie et al., 2008). In contrast, mouse knockout studies with orexin-expressing neurons have shown that orexin may simultaneously promote feeding, energy expenditure and wakefulness (Yamanaka et al., 2003; Lee et al., 2005; Arrigoni et al., 2019). While pleiotropy in homeostatic neural circuits may merely represent an ancestral state conserved across vertebrate lineages, it may also be theorized that hypothalamic neural circuits have been selected to promote a multitude of co-adapted behavioral and physiological responses to facilitate comprehensive behavioral strategies. The pleiotropy of hypothalamic neural circuits appears to extend beyond homeostatic mechanisms, as animal models have also shown that maternal care, which is intricately linked to both feeding and sleep-wake cycles in infancy, is in part mediated by hypothalamic neural circuits in both the mother and the offspring (Chiavegatto et al., 2012; Bridges, 2015). Finally, such pleiotropic effects in hypothalamic control may sometimes be subject to maladaptive effects in humans, as eating disorders, mood and anxiety and schizophrenia may all be associated with dysfunctions of hypothalamic neural circuits (Romano et al., 2016; Bernstein et al., 2019).

Syndromic conditions of hypothalamic dysfunction, particularly neurogenetic conditions involving altered gene dosage, may be hypothesized as extreme manifestations of genetic regulatory mechanisms involved in hypothalamic function. Prader-Willi syndrome, a neurogenetic disorder involving hypothalamic dysfunction (Angulo et al., 2015) highlights alterations in genomic imprinting, an epigenetic regulatory mechanism in which a specific gene is expressed mainly or entirely from one parental allele. Imprinted genes are typically found in evolutionarily conserved clusters where several epigenetic mechanisms including DNA methylation, non-coding RNAs, histone modifications and sequence-specific regulatory proteins function in tandem to regulate imprinted gene expression (Nicholls and Knepper, 2001; Landers et al., 2004; Ivanova and Kelsey, 2011; Marty and Cavaillé, 2019). In PWS, affected individuals carry a genomic alteration, typically due to a meiotic error, which leads to a complete lack of expression for a co-regulated set of ∼20 imprinted genes which are typically expressed solely from paternally inherited alleles in healthy individuals (Cassidy and Driscoll, 2009). The neurobehavioral phenotype of PWS shows evidence for disruption of homeostatic mechanisms regulated in the hypothalamus: PWS involves failure to thrive and comparably increased somnolence particularly in infancy, followed by gradual development of hyperphagia, with lack of satiety and obsessive food-seeking behaviors later in childhood (Haig and Wharton, 2003; Kotler and Haig, 2018). The behavioral phenotypes of PWS also extend beyond altered homeostatic mechanisms, as individuals with PWS typically show mood disturbances, anxiety and frustration towards changes in routine (Schwartz et al., 2021).

Individuals with PWS also show a high prevalence of psychotic symptoms, particularly within the genotype of maternal uniparental disomy (matUPD) which involves both a lack of expression for the affected paternally expressed imprinted genes and increased dosage for the maternally expressed imprinted gene UBE3A. Several independent cohort studies have shown that ∼60% of PWS individuals with the matUPD genotype develop psychotic symptoms in adulthood, and thus, it has been theorized that an imbalance in genomic imprinting *via* increased dosage of UBE3A may predispose PWS matUPD individuals for development of psychosis (Vogels et al., 2004; Soni et al., 2007; Sinnema et al., 2011; Crespi et al., 2018). The hypothesis may thus imply that the matUPD genotype of PWS represents an extreme imbalance towards psychological phenotypes mediated by maternally expressed imprinted genes (Crespi and Badcock, 2008; Kotler and Haig, 2018). Interestingly, it has also been shown that dysfunctions of the hypothalamus are also associated with psychotic disorders (Bernstein et al., 2019; Millard et al., 2022), which might further imply that the extreme psychological phenotype shown with the matUPD genotype of PWS is causal due to hypothalamic dysfunction.

Both within and beyond the scope of PWS, mice models of genomic imprinting further show that paternally expressed genes in particular may take part in regulation of hypothalamic function and simultaneously modify both homeostatic and social behaviors (Ivanova and Kelsey, 2011, also see Supplementary Table S2) In a genome-wide characterization of imprinting in the mouse brain, a significantly larger number of paternally expressed imprinted genes were expressed in the adult hypothalamus, as compared to the number of maternally expressed imprinted genes, while the embryonic mouse brain showed a comparably larger number of active maternally expressed genes (Gregg et al., 2010). A developmental transition in genomic imprinting and hypothalamic control of feeding and somnolence may in part explain the early failure to thrive and the gradual development of hyperphagia in the PWS phenotype (Kotler et al., 2016; Salminen et al., 2019).

Mouse models of PWS also show evidence of pleiotropic phenotypes mediated by altered hypothalamic function, from several lines of evidence. Firstly, SNORD116-deficient model mice show phenotypes of altered food intake (Qi et al., 2015; Polex-Wolf et al., 2018) and sleep-wake cycles (Lassi et al., 2016) and also show a reduced number of orexin-expressing neurons in their lateral hypothalamus as compared to controls (Pace et al., 2020). As orexin-expressing neurons promote wakefulness and food consumption (Arrigoni et al., 2019), these results show an interesting parallel to the PWS phenotype. Lack of expression for MAGEL2 has also been shown to produce a reduction in orexin-expressing neurons, and thus SNORD116 and MAGEL2 may act in a dose-dependent manner in PWS [(Kozlov et al., 2007; Pace et al., 2020)].

Secondly, the MAGEL2 and NDN genes have also been shown to interact with circadian clock proteins in the suprachiasmatic nucleus, affecting phenotypes of circadian rhythmicity (Kozlov et al., 2007; Lu et al., 2020). Circadian rhythmicity affects energy homeostasis and the regulation of sleep-wake cycles *via* diurnally regulated epigenetic cascades affecting gene expression patterns of several thousands of genes downstream (Takahashi, 2017). It has also been shown that lack of expression for SNORD116 alters the diurnal rhythm of gene methylation in model mice, potentially affecting energy homeostasis and epigenetic regulation of several thousands of differentially methylated sites (Coulson et al., 2018).

Thirdly, the social behavioral changes found in PWS may also be altered by a hypothalamic imbalance of oxytocin and oxytocin receptors (Einfeld et al., 2014). Individuals with PWS show reduced numbers of oxytocin-expressing neurons (Swaab et al., 1995; Swaab, 1997) and reduced gene expression for the oxytocin receptor gene, OXTR (Bittel et al., 2007), as compared to controls. However, individuals with PWS also appear to show elevated levels of oxytocin in both blood and cerebrospinal fluids, as compared to controls (Martin et al., 1998; Johnson et al., 2016), which may further indicate a disruption of feedback in oxytocin signaling. Mouse models of PWS, with deletions of MAGEL2 further show that lack of expression for MAGEL2 leads to deficits in social behaviors, but the phenotype may be rescued with post-natal oxytocin treatment (Schaller et al., 2010; Meziane et al., 2015). Thus, evidence from both PWS and other neurogenetic disorders involving hypothalamic dysfunction and animal models of genomic imprinting show that hypothalamic mechanisms may simultaneously affect phenotypes of sleeping, eating, social behaviors and affection, perhaps *via* multiple co-adapted and overlapping neural pathways.

The prevalence for the traits analyzed here within the diagnostic criteria for PWS range from ∼90% for hyperphagic behaviors to 30%–50% for minor criteria such as sleep disturbances (Gunay-Aygun et al., 2001). While individual variation in behavior is known within PWS, the shared genetic origin and further evidence from animal models of hypothalamic function implies that intellectual disability alone cannot account for the observed suite of behaviors in PWS (Whittington and Holland, 2010), thus further implying that phenotypes of social behaviors and affection may also be affected by hypothalamic mechanisms.

Amongst typical human populations, co-adapted pleiotropic phenotypes of hypothalamic function might be manifested as overlapping effects across multiple co-associated phenotypes. As we might suppose that small-effect genetic and epigenetic variation for both imprinted and non-imprinted genes involved in regulation of hypothalamic function may also be circulating among typical human populations, might we also expect to detect non-clinical degrees of co-occurring behavioral variation across a corresponding set of behavioral phenotypes among typically developing individuals? To evaluate this hypothesis, we phenotyped a population of typical individuals for psychological and behavioral traits central to PWS, including autism spectrum cognition, schizotypal cognition, mood, eating, and sleeping, and we estimated the pattern and degree of covariation shown for these traits *via* principal component analysis. While this analysis is limited to behavioral variation and cannot directly implicate specific underlying genetic variation, the co-occurrence of the behavioral variation shown across the relevant phenotypes resembles the set of phenotypes also altered in PWS, thus linking a neurodevelopmental axis highlighted by phenotypical variation in neurodevelopmental disorders to behavioral variation among populations of typical individuals.

## Materials and methods

Questionnaire data was collected between 2019 and 2020 from 589 psychology students (416 females, 170 males, 3 other) at Simon Fraser University and the University of Alberta. To ensure quality of the collected data, the analysis was limited to individuals who had answered every question in the set of questionnaires. After these procedures, a population of 296 individuals (106 males, 190 females) remained. Ethical approval was obtained from the Simon Fraser University and University of Alberta Departments of Research Ethics. The study was advertised in a university questionnaire study portal for psychology students and course credits were offered for participation. The students all provided written informed consent.

A set of demographic variables including biological sex, age, and self-identified ethnicity were collected from all participants. The Autism spectrum quotient (AQ) (Baron-Cohen et al., 2001) was used to characterize the extent of variation for autism spectrum cognitive traits and social behaviors, in ranges relevant to typical human populations while typical variation in schizotypy was assessed with the Schizotypal Personality Questionnaire-Brief Revised (SPQ-BR) (Cohen et al., 2010) questionnaire. Variation in styles of attachment across relationships with the individual’s mother or mother figure, father of father figure, close friend, and romantic partner were characterized with the Experiences in Close Relationships—Relationship Structures Questionnaire (ECR-RS) (Fraley et al., 2011). The Oxford Happiness Questionnaire (Hills and Argyle, 2002) was used in assessing the mood and psychological well-being of the subjects and answers for depression items of the NEO Personality Inventory (NEO-PI) (Costa and McCrae, 1992) were also collected to assess the phenotypes of mood. A modified version of the Pittsburgh Sleep Quality Index (Buysse et al., 1989) without the questions filled out by the partner or roommate was collected to assess the variation in the sleeping habits of the students. The Reduced Morningness-Eveningness Questionnaire (RMEQ) (Adan and Almirall, 1991) was used to assess the ranges of variation in biological rhythmicity. The Dutch Eating Behavior Questionnaire (DEBQ) (van Strien et al., 1986) was used to characterize variation in eating habits and preferences. The questionnaire data was typically collected in early to late afternoon although the timing of the collection was not specifically regulated.

The principal components analysis was conducted in R (stats 3.6.3, 2020) with the princomp function, which calculates eigenvalues and eigenvectors from either a covariance or a correlation matrix of multiple variables to express the covariability within a given data set in principal components. As the set of questionnaires varied highly across their scales, we chose to restrict our analysis to a correlation matrix, which standardizes the effects for each of the variables.

The analysis was further limited to variables considered relevant for hypothalamic function, in respect of the behavioral phenotype of PWS, including total score autism spectrum cognition (AQ), the dimension of cognitive perception in schizotypy (SPQ-BR), attachment-related anxiety in relationship with the individual’s mother (ECR-RS), total score for the Pittsburgh Sleep Quality Index, and the scale of Emotional eating (DEBQ). Two different measures for phenotypes of depression and foul mood were also included: the total score for the Oxford Happiness Questionnaire was reversed by subtracting the individual’s score from the highest score in the data set, creating a scale from 0 to 138 to represent one’s unhappiness in themselves and their circumstances. The scored values for the depression-endorsing items (8 in total) of the NEO personality inventory were also summed up to create an alternative measure of low mood.

A literature review (Supplementary Table S1) was also conducted on known syndromes and case studies of disorders displaying “Prader-Willi–like” phenotypes. Articles were searched using the Web of Science search engine using “Prader-Willi” and the known, hypothalamic phenotypes as search terms. The review was further supplemented by manual searches using references of previously published reviews (Crespi, 2008; Juriaans et al., 2022).

## Results

The analyzed data consisted of a set of 296 individuals for whom complete data was available (190 females and 106 males) with a mean age of 19.6 years (standard deviation of 3.4). The majority of the participants reported their ethnicity as either Caucasian or Asian descent, 46% and 27% respectively. Within the combined population, the reversed score for the OHQ, representing unhappiness, correlated strongly and positively with all of the other traits hypothesized to be relevant for the PWS phenotype of hypothalamic dysfunction while the other traits were also strongly and positively correlated with one another (Table 1). The alternative measure for low mood (NEO-PI depression items) also correlated strongly and positively with traits relevant to the PWS phenotype, indicating a high degree of robustness for the interaction between low mood and PWS-related phenotypes. The correlations, calculated *via* Pearson’s product-moment correlation, remained significant after correction for false discoveries with the Benjamini-Hochberg method. Finally, we find that the phenotypes of Restrained eating and External eating, which are not characteristic of PWS and can thus serve as forms of “control” variables, did not show significant correlations with the reversed OHQ score or the total PSQ score but did correlate strongly with Emotional eating (results not shown).

**TABLE 1:** Pearson moment-product correlations across a of set psychological and behavioral traits deemed to reflect typical degrees of variation in phenotypes relevant to the phenotype of Prader-Willi syndrome. Correlations remained significant after Benjamini-Hochberg correction for false discoveries, as shown in the p adjusted-column.

Phenotype x	Phenotype y	Pearson’s correlation	t	df	p	P adjusted
AQ total score	Cognitive-perceptual scale (SPQ-BR)	0.174	3.028	294	0.003	0.004
	Neo-pi depression items	0.298	5.360	294	1.69^−7^	5.07^−7^
	OHQ-reversed	0.320	5.7940	294	1.77^−8^	7.43^−8^
	Anxiety in mother relationship (ECR-RS)	0.172	2.997	294	0.003	0.004
	PSQ total score	0.165	2.8612	294	0.005	0.005
	Emotional eating (DEBQ)	0.189	3.3009	294	0.001	0.002
Cognitive-perceptual scale (SPQ-BR)	OHQ-reversed	0.241	4.2522	294	2.85^−5^	6.65^−5^
	Neo-pi depression items	0.373	6.8855	294	3.49^−11^	1.83^−10^
	Anxiety in mother relationship (ECR-RS)	0.171	2.9843	294	0.003	0.004
	PSQ total score	0.184	3.2156	294	0.001	0.002
	Emotional eating (DEBQ)	0.222	3.9031	294	0.0001	2.25^−4^
OHQ-reversed	Anxiety in mother relationship (ECR-RS)	0.243	4.3031	294	2.30^−5^	6.04^−5^
	Neo-pi depression items	0.675	15.693	294	2.20^−16^	2.31^−15^
	PSQ total score	0.458	8.8298	294	2.20^−16^	2.31^−15^
	Emotional eating (DEBQ)	0.238	4.2057	294	3.46^−5^	7.27^−5^
Neo-pi depression items	Anxiety in mother relationship (ECR-RS)	0.212	3.7263	294	2.33^−4^	4.08^−4^
	PSQ total score	0.447	8.569	294	6.00^−16^	4.20^−15^
	Emotional eating (DEBQ)	0.311	5.6199	294	4.44^−8^	1.55^−7^
Anxiety in mother relationship (ECR-RS)	PSQ total score	0.145	2.5041	294	0.013	0.0135
	Emotional eating (DEBQ)	0.119	2.0463	294	0.042	0.0416

The first principal component (PC1) accounted for ∼35% of the total variance within the population and included positive loadings for all of the PWS-related traits (see Tables 2, 3). PC2 accounted for ∼15% of the variance and included a negative loading for the reversed OHQ scores, and the total PSQI score, while showing positive loadings for the other traits (See Table 4). The three largest principal components accounted for 65% of the total variance within the combined population. Alternative analyses using the NEO-PI depression items instead of the reversed OHQ score performed in a similar manner, but PC2 also included a negative loading for Emotional eating (DEBQ) (See Tables 3, 4). Analyses divided by sex showed minor differences to the multivariate structures shown for the combined population. The proportion of variance covered by the largest principal component is somewhat larger (∼42% males, 39% for females %) compared to combined population. The largest principal component was similarly characterized by positive loadings for all six variables with both sexes. However, females uniquely show a negative loading for anxiety in mother relationships for PC2, while the trait loaded positively on PC2 with males (Tables 5, 6).

**TABLE 2:** Principal components analysis of the variation for the reversed OHQ score and phenotypes in ranges of typical variation for schizotypy, autism spectrum cognition, attachment-related anxiety, sleep and emotional eating, as also shown in Table 1. The principal components were calculated from eigenvalues on the correlation matrix, only the three largest principal components are shown for each analysis.

Both-OHQ	PCA	Variance proportion	Cumulative proportion	St.dev
(*N* = 296)	PC1	0.352	0.352	1.452
	PC2	0.151	0.502	0.951
	PC3	0.149	0.651	0.945
Females				
(*N* = 190)	PC1	0.330	0.330	1.406
	PC2	0.190	0.520	1.068
	PC3	0.143	0.662	0.925
Males				
(*N* = 106)	PC1	0.378	0.378	1.507
	PC2	0.171	0.549	1.013
	PC3	0.161	0.710	0.983

**TABLE 3:** Alternate principal components analysis replacing the reversed OHQ score with a sum of the depression-endorsing items of the NEO Personality inventory.

Both-neo-pi	PCA	Variance proportion	Cumulative proportion	St.dev
(*N* = 296)	PC1	0.361	0.361	1.473
	PC2	0.152	0.513	0.954
	PC3	0.142	0.655	0.921
Females				
(*N* = 190)	PC1	0.333	0.333	1.413
	PC2	0.182	0.514	1.044
	PC3	0.144	0.658	0.928
Males				
(*N* = 106)	PC1	0.392	0.392	1.534
	PC2	0.170	0.563	1.011
	PC3	0.164	0.727	0.991

**TABLE 4:** Loadings for each variable across the three largest principal components in the analyses for the combined population (*N* = 296).

Both-OHQ	Variable	PC1	PC2	PC3
*N* = 296	Total AQ score	0.38837	0.13650	0.16058
	Cognitive-perceptual	0.37179	0.48263	−0.23554
	Unhappiness (OHQ)	0.52514	−0.35915	0.05906
	Anxiety in mother relationship (ECR-RS)	0.32930	0.32214	0.75914
	Total PSQI score	0.44100	−0.62766	−0.10832
	Emotional eating (DEBQ)	0.36366	0.34888	−0.57202
**Both-neo-pi**		**PC1**	**PC2**	**PC3**
	Total AQ score	0.36316	0.31957	0.39689
	Cognitive-perceptual	0.40827	−0.03523	0.17619
	NEO-PI depression items	0.53917	−0.18974	−0.15807
	Anxiety in mother relationship (ECR-RS)	0.30164	0.81754	−0.28326
	Total PSQI score	0.42017	−0.36007	−0.61947
	Emotional eating (DEBQ)	0.37841	−0.25021	0.56787

**TABLE 5:** Loadings for each variable across the three largest principal components in the subdivided analyses limited to females (*N* = 190).

Females-OHQ		PC1	PC2	PC3
	Total AQ score	0.39672	0.11636	0.38052
	Cognitive-perceptual	0.36298	0.47936	0.01189
	Unhappiness (OHQ)	0.54545	−0.23769	−0.24759
	Anxiety in mother relationship (ECR-RS)	0.36903	−0.26135	0.70616
	Total PSQI score	0.46599	−0.35988	−0.52000
	Emotional eating (DEBQ)	0.24498	0.70877	−0.15718
**Females-neo-pi**		**PC1**	**PC2**	**PC3**
	Total AQ score	0.39115	0.01455	0.57188
	Cognitive-perceptual	0.42595	0.37065	−0.11419
	NEO-PI depression items	0.54395	−0.09257	−0.27552
	Anxiety in mother relationship (ECR-RS)	0.32982	−0.42105	0.55872
	Total PSQI score	0.41995	−0.42608	−0.52044
	Emotional eating (DEBQ)	0.29077	0.70357	0.03127

**TABLE 6:** Loadings for each variable across the three largest principal components in the subdivided analyses limited to males (*N* = 106).

Males-OHQ		PC1	PC2	PC3
	Total AQ score	0.42474	0.07000	0.11739
	Cognitive-perceptual	0.30949	0.45395	0.66039
	Unhappiness (OHQ)	0.50714	−0.16530	0.16649
	Anxiety in mother relationship (ECR-RS)	0.31433	0.65815	−0.44884
	Total PSQI score	0.40077	−0.55053	0.13253
	Emotional eating (DEBQ)	0.45519	−0.15958	−0.55078
**Males-neo-pi**		**PC1**	**PC2**	**PC3**
	Total AQ score	0.39033	0.10859	0.02299
	Cognitive-perceptual	0.33545	0.35113	0.70654
	Unhappiness (OHQ)	0.52951	−0.15288	0.18498
	Anxiety in mother relationship (ECR-RS)	0.30336	0.69320	−0.36659
	Total PSQI score	0.40532	−0.59014	0.05579
	Emotional eating (DEBQ)	0.44545	−0.11295	−0.57320

The covariability shown for the diverse set psychological and physiological traits analyzed may indicate that typical variation for phenotypes of schizotypy, attachment, mood, sleep and eating habits are partly mediated by a hypothalamic axis of neural regulation. The variation for each of the traits correlated positively with one another, showing for example that high degrees of schizotypy were positively correlated with less secure attachment, comparably higher tendencies for emotional eating and comparably more severe sleep problems. In addition, the reversed OHQ, which corresponds to one’s unhappiness and lack for sense of fulfilment, showed a strongly positive and significant correlation with the other traits as shown in Table 1, while the NEO-PI depression items performed similarly, indicating robustness for the effect observed.

To further explore if hypothalamic dysfunctions may simultaneously alter covarying sets of behavioral phenotypes, we reviewed a large body of literature, focusing on conditions where individuals have been reported to show behavioral phenotypes partly overlapping with the set of behavioral phenotypes also altered in PWS. As also shown in Supplementary Table S1, a set of clinical conditions and differential diagnoses or “Prader-Willi–like” syndromes can be recognized, whereby individuals present with a set of phenotypes resembling some of those of PWS yet lack the methylation alteration, maternal uniparental disomy, or large deletion diagnostic of this syndrome.

The main findings from this review were threefold: Firstly, a number of imprinted and non-imprinted genes that are implicated in “Prader-Willi–like” syndromes, may also interact directly with the imprinted loci involved in PWS. The IPW non-coding RNA gene, located within the 15q11-q13 genomic region has been found to also interact with transcription of the imprinted genes within DLK1-DIO3 imprinted locus, which in turn is affected in the Kagami-Ogata and Temple syndromes. It has been proposed that such crosstalk between imprinted loci may reflect common evolutionary origins and co-adaptation between the two or perhaps several imprinted loci (Stelzer et al., 2014). Secondly, while phenotypical presentations showed variation on the individual level due to varying genomic alterations across cases, rather than resembling PWS with one specific phenotype, PWS-like conditions tend to show pleiotropic sets of behavioral phenotypes, partly resembling the full set of phenotypes similarly also altered in PWS. Thirdly, mouse and cell model studies focusing on potential neural mechanisms of the candidate genes involved in PWS-like conditions further highlight both hypothalamic mechanisms and mechanisms related to the excitation-inhibition balance of the brain in particular. This review thus provides evidence that a diverse set of covarying phenotypes are altered in both PWS and the “Prader-Willi–like” syndromes and dysfunctions of hypothalamic mechanisms may be further implicated in these conditions *via* alterations of both imprinted and non-imprinted genes, further implying a hypothalamic axis of neural and genetic effects on human behavior that are not wholly separate from one another, such that alterations on a singular regulatory mechanism may have several pleiotropic phenotypic effects downstream.

## Discussion

In this study, we have collected and analyzed questionnaire data from a population of healthy individuals to evaluate if a set of PWS-associated psychological and physiological traits relevant to hypothalamic function, would also show a corresponding pattern of covariation in the ranges of typical variation found among healthy individuals. The questionnaire data showed that phenotypes of sleeping and emotional eating, governed in part by the hypothalamus, appear to co-vary with phenotypes of schizotypy, mood and anxiety-related aspects of attachment. The overlap of such a diverse set of physiological and behavioral traits in part mediated by the hypothalamus may imply an evolutionary history whereby neural circuits responsible for regulation of hunger, satiety and biorhythmicity were co-opted to jointly regulate affection and social behaviors in the context of maternal care.

The genomic conflict theory of imprinting (Haig and Wharton, 2003; Kotler and Haig, 2018) which states that genomic imprinting has evolved due to a conflict in resource allocation between maternally and paternally inherited alleles, provides a useful explanation for why hypothalamic traits might be mediated by genomic imprinting to regulate phenotypes of sleep, feeding, and attachment, as the phenotypes of these traits among the offspring may be expected to jointly affect the maternal investment of care into their offspring. Thus, alleles of paternally expressed imprinted genes, which may not share their genotype with other members of the offspring due to the statistical probability for mixed parenthood, may have been selected to favor a hypothalamic phenotype comparably more demanding towards the mother and might thus be under selection for effects that involve spending a larger proportion of the subjective day active while also demanding more affection and food from their mother. By contrast, PWS phenotypes, which are due to lack of expression for such paternally expressed imprinted genes and thus involve a bias in gene dosage towards expression of maternally inherited imprinted genes, would be expected to reflect an extreme manifestation of mechanisms that select for a comparably more equal distribution of maternal investment across offspring, and less individually demanding phenotypes with increased somnolence, reduced affection and reduced food intake in infancy, when the child is most dependent on their mother for sustenance (Kotler and Haig, 2018; Salminen et al., 2019). As healthy individuals may also show less extreme, non-clinical variation for gene expression of imprinted, and non-imprinted genes involved in hypothalamic function, co-variation across a corresponding set of behavioral phenotypes may also be shown, due to neural and genetic regulatory mechanisms that underlie typical function of these phenotypes.

In addition to the altered phenotypes of PWS, several lines of evidence also show that hypothalamic mechanisms may also modify phenotypes of social behavior. Firstly, evidence for structural abnormalities and functional deficits in hypothalamic circuits has also been shown in schizophrenia (Bernstein et al., 2019; Millard et al., 2022). Secondly, oxytocin pathways have been consistently associated with bonding between the mother and offspring (Scatliffe et al., 2019) but maladaptive effects of oxytocin pathways in social behavior may be highlighted in studies showing that variation in oxytocin function may be associated with schizotypy, autism spectrum disorders and depression (Feldman et al., 2016). In PWS, variation of sleep phenotypes has also been significantly associated with psychosis-risk symptoms, linking a correlate of hypothalamic dysfunction to psychotic symptomatology in PWS (O’Hora et al., 2022). A small number of subjects both with and without PWS have also been reported to develop manic symptoms or panic attacks due to hypothalamic stimulation with electrodes, which might similarly suggest an association between hypothalamic dysfunction and psychotic symptoms (Franco et al., 2018; Elias et al., 2020).

We have also reviewed a large body of literature (Supplementary Table S1) on disorders displaying phenotypes implying of hypothalamic dysfunction *via* altered phenotypes of food intake, sleep or both. Our approach was primarily guided by the phenotype of PWS, which involves a lack of expression for multiple paternally expressed imprinted genes and shows a highly pleiotropic phenotype of hypothalamic dysfunction with altered sleep, feeding, affection, mood and social cognition (Angulo et al., 2015; Crespi et al., 2018; Salminen et al., 2019). While the set of disorders resembling PWS is varied and involves dysfunctions of both imprinted and non-imprinted genes (Juriaans et al., 2022), we also find that candidate genes that are associated with hypothalamic phenotypes also tend to be associated with altered behavior among these disorders. Of particular note are phenotypes shown in Kleefstra syndrome, which involves hypotonia, food seeking behaviors and obesity with a sleep phenotype of repeated night awakenings and hyperactivity, while a limited number of cases also involve phenotypes of frustration and temper tantrums in childhood and psychosis, bipolar disorders and apathy among adult cases (Verhoeven et al., 2011; Kleefstra et al., 2012; Adam and Isles, 2017; De Taevernier et al., 2021). Thus, the syndrome features behavioral phenotypes that resemble phenotypes present in PWS and physiological phenotypes mediated by the hypothalamus which both resemble PWS in part while also showing opposite tendencies for altered sleep phenotypes. We also find, as noted in previous work (reviewed in Ivanova and Kelsey, 2011) that paternally expressed imprinted genes appear to be particularly active in the hypothalamus as is also shown by the genome-wide pattern genomic imprinting in the mouse brain (Gregg et al., 2010). The mouse hypothalamus showed a larger number of active paternally expressed imprinted genes as compared to maternally expressed imprinted genes, a pattern opposite to that of the embryonic mouse brain, which may thus highlight the hypothalamus as a hotspot of paternal-origin allelic expression (Gregg et al., 2010; Ivanova and Kelsey, 2011). Paternally expressed imprinted genes expressed in the hypothalamus also show phenotypes relevant to allocation of maternal resources in model mice: In particular, a large of paternally expressed imprinted genes were active in hypothalamic neural circuits and were also associated with altered phenotypes relevant to maternal care including sleep, feeding, parenting behaviors, anxiety or social cognition (Supplementary Table S2 for references), reflecting the affected phenotypes also shown PWS.

Our findings may also be evaluated in the context of previous behavioral studies which have also indicated correlations between pairs of behavioral traits shown in our questionnaire data. Firstly, as PWS has been found to show varying incidence rates for ASDs in prior studies (Dykens et al., 2011), a measure of autism spectrum cognition was also included in our analysis. While our data showed degree of covariation between typical variation of autism spectrum cognition and variation of other phenotypes deemed to be relevant for the PWS phenotype, earlier questionnaire studies have also shown that both clinical and non-clinical variation in behavioral traits that extend towards the spectrums of autism and schizotypy tend to co-occur or correlate positively with one another to some degree (Dinsdale et al., 2013; Kincaid et al., 2017; Sampson et al., 2021). Studies among individuals with clinical forms of psychosis also appear to show elevated rates of ASDs as compared to the typical human populations and autism–like behavioral traits are similarly precent in high percentages, varying between 9%–61% of individuals diagnosed with psychotic symptoms (Kincaid et al., 2017). It has been traditionally theorized that the co-occurrence of schizotypy and autism spectrum traits may be partly due to differential diagnosis and overlapping behavioral phenotypes, particularly between negative psychotic symptoms and social deficits in ASDs [reviewed in (Kincaid et al., 2017)]. However, studies concerning non-clinical variation in schizotypy and autism spectrum traits have shown correlations between both negative and positive schizotypy and autism spectrum traits (Dinsdale et al., 2013; Kincaid et al., 2017).

In the data analyzed here, the cognitive-perceptual dimension of schizotypy, as measured *via* the SPQ-BR questionnaire may be interpreted to represent healthy variation within a spectrum of behaviors that extends towards the positive symptoms of schizophrenia, which include ideas of reference (“I often feel that others have it in for me”), magical thinking (“Have you ever felt that you are communicating with another person telepathically?”), and unusual perceptions (“Are your thoughts sometimes so strong that you can almost hear them?”). Both the cognitive-perceptual scale and SPQ-BR total scores were significantly and positively correlated with AQ total scores in our data. Hence, in the context of our current study and earlier studies, it is not entirely clear whether genetic variation and neural mechanisms prevalent in ASDs may 1) function as risk factors for development of psychotic symptoms later in life, or 2) if the two disorders overlap in phenotype only but are mediated by separate genetic and neural mechanisms. Considering the psychological phenotypes of hypothalamic dysfunction shown in both PWS and the altered phenotypes of attachment and social behavior shown in mouse models with deletions of paternally expressed imprinted genes (Supplementary Table S2), it may be further theorized that genetic variation for imprinted genes and genes related hypothalamic function might simultaneously predispose individuals to covarying effects for altered phenotypes of social behaviors and insecure attachment, which may contribute towards variation involving overlap of social deficits between schizotypy and autism spectrum cognition. Relevant to this hypothesis and in similarity to covariation shown in our results, variation in the continuum of paranoia has been shown to be positively correlated with insecure and anxious attachment styles (Korver-Nieberg et al., 2014; Murphy et al., 2020), which has been further theorized to indicate that dysfunctional Theory of Mind (ToM) cognition may predispose individuals to insecure attachment *via* overly negative models of how others might perceive oneself (Pietromonaco et al., 2000).

Correlations between sleep problems, typically measured by the PSQI questionnaire have also been observed to correlate positively and significantly with eating disorders and emotional eating in prior studies (Soares et al., 2011; Trace et al., 2012; Saleh-Ghadimi et al., 2019) It has also been previously theorized that such correlations might indicate neural mechanisms in the hypothalamic-pituitary axis jointly regulating sleep and feeding might underlie the correlations observed between sleep problems and disordered eating (Trace et al., 2012).

Our analyses are affected by three main limitations that must be considered in relation to the results shown. Firstly, our analyses are limited to self-evaluated behavioral variation and cannot directly implicate specific underlying genetic variation within the study population. Secondly, the set of behavioral phenotypes studied is intended to represent a set of evolutionarily relevant correlates in ranges of typical function rather than traits directly and fully comparable to PWS phenotypes. As PWS typically involves severe hyperphagia with lack of satiety and food-related obsessive behaviors (Dykens et al., 2007), the observed behaviors may also lack a clear correlate within ranges of typical eating behaviors. For the purposes of our study, we have primarily considered the phenotype of Emotional eating, which involves overeating due to emotional responses and may thus partially reflect hypothalamic function rather than external eating, which involves overeating due to how appetizing the food seems or restrained eating, which may involve a conscious decision to restrict eating (van Strien et al., 1986). Thirdly, individual neural mechanisms of hypothalamic function may only be considered from a theoretical viewpoint, as we lack experimental evidence to assign any specific mechanism of neural function to the behavioral phenotypes shown in the study population.

Apart from the aforementioned limitations, it may also be questioned whether genetic polymorphisms affecting hypothalamic function are circulating among typically developed human populations. We note that as shown within a population of healthy human donors from Miami Brain Bank, published under the Genotype-Tissue Expression Portal project (GTEx Portal genome browser, accessed on 7th October 2022), the long non-coding RNA, PWRN1 shows a high number of expression quantitative trait loci SNPs for gene expression differences in hypothalamic tissues, located both within and in the promoter regions of the gene. PWRN1 may also be expressed in the brain as a part of the SNURF-SNRPN (SNHG14) RNA transcript (Chamberlain, 2013). Furthermore, the SNORD116 snoRNA, which is the only single locus implicated in the main behavioral phenotypes of PWS (reviewed in Hassan and Butler, 2016) is also processed from the introns of the SNURF-SNRPN transcript (Runte et al., 2001), and hence, polymorphisms affecting expression of PWRN1 might also indirectly affect the expression of SNORD116. While differences in gene expression within hypothalamic tissues are not in themselves indicative of behavioral variation, these polymorphisms nevertheless showcase a theoretical mechanism for non-clinical variation in regulation of hypothalamic function.

In conclusion, the co-occurring set of physiological and psychological phenotypes shown in PWS and other “Prader-Willi–like” neurogenetic syndromes implies that of hypothalamic neural circuits and their genetic regulatory mechanisms may have become co-adapted to jointly regulate phenotypes of sleep, food intake and social behaviors in the context of maternal investment. In particular, we highlight the sets of physiological and behavioral phenotypes associated with 1) oxytocin- and 2) orexin-expressing neurons in animal model studies and neurogenetic disorders. Alterations in function of oxytocin neurons have been independently associated with alterations in maternal care (Bridges, 2015), feeding (Romano et al., 2020) and psychological functioning (Bernstein et al., 2019) while alterations in orexin-expressing neurons have similarly been associated with alterations in sleep and feeding (Pace et al., 2020) as well as alterations of social behaviors (Harris et al., 2020), pair-bonding, and parenting behaviors (Donlin et al., 2014; Bridges, 2015). Here, we show that behavioral variation present among healthy individuals shows evidence for patterns of co-variation for a set of behavioral and physiological phenotypes which may represent a non-clinical extension of neural functions in part mediated by hypothalamic neural circuits. Thus, alterations in regulation of hypothalamic circuits might predispose individuals to a diverse set of co-occurring behavioral and psychological alterations, which should be considered in both research of psychological and physiological disorders and counseling among healthy individuals.

## Data Availability

The raw data supporting the conclusion of this article will be made available by the authors, without undue reservation.

## Supplementary material

SUPPLEMENTARY TABLE S1 Disorders showing pleiotropic behavioral phenotypes resembling the phenotype hypothalamic dysfunction in Prader-Willi syndrome. The affected genes and phenotypes of known cases may vary within the syndromes and the neural mechanisms listed are limited to postulation, based on the genotypes of known cases and relevant mouse model studies.

SUPPLEMENTARY TABLE S2 Paternally expressed imprinted genes active in hypothalamus showing phenotypes relevant to affection and maternal investment in mouse model studies and neurogenetic disorders.
