# Supplementary material for: Do the diverse phenotypes of Prader-Willi syndrome reflect extremes of covariation in typical populations?
Source: Front Genet. 2022 Nov 24;13:1041943. doi: 10.3389/fgene.2022.1041943 (PMC9731222; doi:10.3389/fgene.2022.1041943)
Supplement: Supplementary file 1 [file Table1.DOCX]

| Disorder | Genetic alteration | Proposed neural mechanism | Associated behavioral phenotypes |
| --- | --- | --- | --- |
| Prader-Willi syndrome | Loss of paternally expressed genes in 15q11-q13 chromosome region (1) | Hypothalamic dysfunction (2), SNORD116 may in part in regulate the expression of thousands of genes in the brain (3) | High prevalence of psychotic spectrum conditions (4), temper tantrums, mood lability, depression (5), reduced sleep latency (6), failure to thrive, hyperphagia in late childhood (7), skin picking and high pain tolerance (8) |
| Schaaf-Yang syndrome | MAGEL2 non-sense mutations or microdeletions (9). | MAGEL2 involved in development of oxytocin-expressing neurons (10), leptin-induced appetite reduction response in hypothalamus (11). | Hypotonia, feeding problems, High prevalence of autism spectrum behaviors (as compared to PWS) (9), hyperphagia and psychotic features in adulthood, disturbed sleep cycle with daytime fatigue (12). |
| Temple syndrome, DLK1/MEG3 imprinted region | Lack of expression for paternally expressed genes DLK1, DIO3 and RTL1 (13) | DLK1 expressed in hypothalamus (14), PWS-associated non-coding RNA IPW may also regulate imprinted gene expression in the DLK1-DIO3 locus (15) | Hypotonia, feeding difficulties during infancy, mild obesity and increased appetite reported with ~ ½ of cases, emotional problems with a small number of cases (13,16) |
| Del 1p36 syndrome | Haploinsufficiency for genes within 1p36 chromosome region, including MMP23A/B GABRD and SKI (17) | GABRD deletion may model post-partum depression in mice (18), involved in stress response in hypothalamus (19) | Temper outbursts and behavioral problems, hypotonia and feeding difficulties , hyperphagia at ~ 3 years of age, skin picking or pinching with high pain tolerance reported with PWS-like individuals (20, 17) |
| 2p25(2pter) deletion | Haploinsufficiency for genes within 2p25, MYT1L is located within the SRO for known cases (21). | MYT1L ortholog knockout in a zebrafish model showed misdevelopment of the neuroendocrine hypothalamus (22). | Hypotonia, early development of obesity with ~ 50 % of cases, hyperphagia, mood changes, intolerance to frustration, temper tantrums, anxiety, hyperactivity, self-harming behavior, difficulties with falling asleep, night awakenings with ~ 33 % of cases (23) |
| 2q37 deletion syndrome | Haploinsufficiency of genes within 2q37, PER2, HDAC4 are listed as a candidate scene for PWS-like phenotypes (24, 25). | Clock-gene PER2 expressed in suprachiasmatic nucleus (26) and HDAC4 expressed in hypothalamus (27) | Hypotonia, obesity, poor feeding, temper tantrums, self-mutilating behaviors (24, 25, 28) |
| 3p24.1-p26.2 duplication | Duplication including OXTR, PPARG1, SLC6A1, SLC6A11 (29) | Oxytocin expressed primarily in hypothalamus, involved in maternal behaviors, empathy and trust (30), expression of OXTR reduced in PWS individuals (31) | Hypotonia, obesity in without food foraging, frustration, stubbornness, anxiety (29, 32) |
| 6q16.2 deletion | SIM1, MCHR2 haploinsufficiency  (33). | SIM1 haploinsufficiency causes obesity and hyperphagia in a mouse model, reductions in oxytocin- and vasopressin-producing neurons in the hypothalamus (34). | Hypotonia, obesity, hyperphagia, emotional instability, fits of anger (33, 35) |
| 9q34 deletion, Kleefstra syndrome (KS) | Haploinsufficiency for EHMT1 likely responsible for developmental delay/hypotonia (36) | Neurodegeneration due to impaired maintenance of DNA methylation has proposed as causal to apathy and psychosis in KS (37). | Hypotonia, food seeking behaviors and obesity present in both intragenic EHMT1 mutations and larger deletions (~ 45 % of all cases), repeated night awakenings, hyperactivity, frustration, temper tantrums in childhood. Psychosis, bipolar disorders and apathy reported with adult cases (36, 38, 39) |
| 10q26.2 deletion | Haploinsufficiency for genes within the 10q genomic region overlapping with ~100 genes, DOCK1 has hypothesized as a candidate gene (40, 41 ,42). | DOCK1, INSYN2A, NPS within a proposed critical region, involved in nervous system development and function (43) NPS also expressed in hypothalamus, involved in regulation of anxiety –like behaviors (44). | Hypotonia, developmental delay, hyperactive, agitated behaviors with similarities to oppositional defiant disorder, disruptive and affectionate behavior, bipolar disorder –like symptoms (40, 45) |
| 15q26 deletion | Haploinsufficiency for genes within 15q26, CHD2 and RGMA proposed as candidate genes (46) point mutations of CHD2 associated with epilepsy and ASD –like behaviors (47). | Haploinsufficient mouse model for CHD2 shows changes excitatory and inhibitory synaptic function (48). Partly opposite changes in excitatory and inhibitory balance have been proposed to be involved development of both ASDs and psychotic disorders (49). | Hypotonia, truncal obesity with over-eating, anxious disorder in puberty and adulthood, temper tantrums related to changes in daily routine (46) ataxia, ASD and Angelman –like behaviors at 6 years of age (50) Psychotic disorders in adulthood reported on four individuals with CHD2 mutations (51, 52, 53). |
| 15q duplication (PWAS region) | Duplication of 15q11-q13 region, ~80 % maternal inverted duplication or isodicentric chr 15, increased dosage of UBE3A, GABRB3, OCA2, HERC2 (54) | Increased dosage of UBE3A has been theorized to contribute an increased susceptibility for psychotic disoders both in PWS (55) and with 15q11-q13 duplications (56), UBE3A may affect inhibitory balance via GABAercic neurons (57). | Hypotonia, developmental delay, ASD- like behaviors, anxiety, sleep disturbance, self-aggression. Overweight was also reported 2/26 cases (54). Familial cases carrying a UBE3A duplication showed a history of learning difficulties, ASD –like behaviors and psychotic disorders (58). |
| 19p13.3 deletion | Haploinsufficiency for genes including ZBTB7A, PIAS4, MAP2K2(59, 60), point mutations of both ZBTB7A and PIAS4 are associated with macrocephaly and developmental delay 61, 62). | ZBTB7A is expressed in developing white matter (63), PIAS4 is an E3 ubiquitin ligase involved in Wnt –signalling (64, 65). | Obesity, developmental delay, self-aggression, temper tantrums (59). Deletions with ZBTB7A, MAP2K2 and PIAS4 involve hypotonia, feeding problems and developmental delay (60). |
| Fragile X syndrome | Lack of expression for the X-linked FMR1 gene, due to excessive amounts of CGG repeats in FMR1 ‘5 exon (66) | CYFIP1 interacts directly FMR1, expression levels of CYFIP1 severely reduced in FXS individuals with PWS-like phenotypes (67). CYFIP1 may be also involved in the regulation of GABAergic excitation-inhibition balance (68). | FXS individuals with PWS –like phenotype typically present with obesity, early onset of hyperphagia, food-seeking behavior, temper tantrums and obsessive behaviors, weight increase without change in diet also reported (66, 67, 69). |
| Smith-Magenis syndrome (SMS) | Haploinsufficiency for genes within 17p11.2 and point mutations within RAI1 (70). | Mouse model with haploinsufficiency for RAI1 shows reduced expression of BDNF in hypothalamus with increased food intake as compared to controls (71), and social deficits in interactions with unfamiliar control mice (72). | Hypotonia, increased meal size, (individuals are able to feel full) temper tantrums, aggressive behaviors, attention-seeking, self-injury, difficulties falling asleep. (70, 73). High prevalence of ASD symptoms with a reversed gender ratio as compared to typical populations (74). |
| Kleine-levin syndrome | Idiopathic, may be associated with viral or post-infectious autoimmune encephalitis with primary impact on the hypothalamus (75) | TRANK1 polymorphisms may predispose individuals to KLS, involved in circadian regulation, also associated with psychotic disorders (76). Some cases may involve hypothalamic dysfunction or lesions (77). | Hypersomnia, compulsive eating behavior, apathy, derealization, cognitive disorders resembling psychotic spectrum (75). |
| Hyperphagic short stature syndrome, psychosocial short stature | Idiopathic, associated with emotional stress (78, 79). | Growth hormone insufficiency, has been hypothesized to be associated with hypothalamic dysfunction 78, 80). | Hyperphagia, developmental delay, self-injury, sleep disturbance, anxious, oppositional behaviors (78), psychosis reported in one known case (79). |

References in table: (1) (Nicholls and Knepper, 2001), (2) (Angulo et al., 2015), (3) (Coulson et al., 2018) (4) (Crespi et al., 2018), (5) (Feighan et al., 2020), (6) (Salminen et al., 2019), (7) (Kotler et al., 2016), (8) (Cassidy et al., 2012), (9) (McCarthy et al., 2018), (10) (Ates et al., 2019), (11) (Mercer et al., 2013), (12) (Marbach et al., 2020), (13) (Ioannides et al., 2014), (14) (Persson-Augner et al., 2014), (15) (Stelzer et al., 2014), (16) (Kagami et al., 2017), (17) (D’Angelo et al., 2010), (18) (Maguire and Mody, 2008), (19) (Lee et al., 2014) (20), (D’Angelo et al., 2006), (21) (Stevens et al., 2011) (22) (Blanchet et al., 2017), (23) (Coursimault et al., 2022), (24) (Leroy et al., 2013), (25) (Le et al., 2019), (26) (Mielcarek et al., 2015), (27) (Kim M et al., 2018) (28) (Villavicencio-Lorini et al., 2012) (29) (Natera-de Benito et al., 2014) (30) (MacDonald and MacDonald, 2010) (31) (Bittel et al., 2007) (32) (Bittel et al., 2006), (33) (Khattabi et al., 2015) (34) (Duplan et al., 2009) (35) (Varela et al., 2006), (36) (Kleefstra et al., 2009), (37) (Adam and Isles, 2017), (38) (Verhoeven et al., 2011), (39) (De Taevernier et al., 2021), (40) (Yatsenko et al., 2009), (41) (Faria et al., 2016), (42) (Lin et al., 2016), (43) (Cherik et al., 2021), (44) (Grund and Neumann, 2019), (45) (Courtens et al., 2006), (46) (Courage et al., 2014), (47) (De Maria et al., 2022), (48) (Kim YJ et al., 2018) (49) (Canitano and Pallagrosi, 2017) (50) (Capelli et al., 2012) (51) (Verhoeven et al., 2016), (52) (Bernardo et al., 2017), (53) (Thygesen et al., 2018), (54) (Al Ageeli et al., 2014), (55) (Soni et al., 2007), (56) (Ingason et al., 2011), (57) (Lopez et al., 2019), (58) (Noor et al., 2015) (59) (de Smith et al., 2011), (60) (Nevado et al., 2015), (61) (Ohishi et al., 2020), (62) (Tenorio et al., 2020), (63) (Dobson et al., 2012), (64) (Burn et al., 2011), (65) (Miller et al., 2013), (66) (de Vries et al., 1993), (67) (Nowicki et al., 2007), (68) (Davenport et al., 2019), (69) (Schrander‐Stumpel et al., 1994), (70) (Edelman et al., 2007), (71) (Burns et al., 2010), (72) (Rao et al., 2017), (73) (Alaimo et al., 2015), (74) (Nag et al., 2018), (75) (Arnulf et al., 2005), (76), (Ambati et al., 2021), (77) (Kostić et al., 1998), (78) (Gilmour et al., 2001), (79) (Wattchow et al., 2015), (80) (Gilmour and Skuse, 1999)

Supplementary table 1. Disorders showing pleiotropic behavioral phenotypes resembling the phenotype hypothalamic dysfunction in Prader-Willi syndrome. The affected genes and phenotypes of known cases may vary within the syndromes and the neural mechanisms listed are limited to postulation, based on the genotypes of known cases and relevant mouse model studies.

Adam, M. A., and Isles, A. R. (2017). EHMT1/GLP; Biochemical Function and Association with Brain Disorders. *Epigenomes 2017, Vol. 1, Page 15* 1, 15. doi:10.3390/EPIGENOMES1030015.

Al Ageeli, E., Drunat, S., Delanoë, C., Perrin, L., Baumann, C., Capri, Y., et al. (2014). Duplication of the 15q11-q13 region: Clinical and genetic study of 30 new cases. *Eur. J. Med. Genet.* 57, 5–14. doi:10.1016/J.EJMG.2013.10.008.

Alaimo, J. T., Barton, L. V., Mullegama, S. V., Wills, R. D., Foster, R. H., and Elsea, S. H. (2015). Individuals with Smith-Magenis syndrome display profound neurodevelopmental behavioral deficiencies and exhibit food-related behaviors equivalent to Prader-Willi syndrome. *Res. Dev. Disabil.* 47, 27–38. doi:10.1016/J.RIDD.2015.08.011.

Ambati, A., Hillary, R., Leu-Semenescu, S., Ollila, H. M., Lin, L., During, E. H., et al. (2021). Kleine-Levin syndrome is associated with birth difficulties and genetic variants in the TRANK1 gene loci. *Proc. Natl. Acad. Sci. U. S. A.* 118. doi:10.1073/PNAS.2005753118.

Angulo, M. A., Butler, M. G., and Cataletto, M. E. (2015). Prader-Willi syndrome: A review of clinical, genetic, and endocrine findings. *J. Endocrinol. Invest.* 38, 1249–1263. doi:10.1007/s40618-015-0312-9.

Arnulf, I., Zeitzer, J. M., File, J., Farber, N., and Mignot, E. (2005). Kleine–Levin syndrome: a systematic review of 186 cases in the literature. *Brain* 128, 2763–2776. doi:10.1093/BRAIN/AWH620.

Ates, T., Oncul, M., Dilsiz, P., Topcu, I. C., Civas, C. C., Alp, M. I., et al. (2019). Inactivation of Magel2 suppresses oxytocin neurons through synaptic excitation-inhibition imbalance. *Neurobiol. Dis.* 121, 58–64. doi:10.1016/J.NBD.2018.09.017.

Bernardo, P., Galletta, D., Iasevoli, F., D’Ambrosio, L., Troisi, S., Gennaro, E., et al. (2017). CHD2 mutations: Only epilepsy? Description of cognitive and behavioral profile in a case with a new mutation. *Seizure* 51, 186–189. doi:10.1016/j.seizure.2017.09.001.

Bittel, D. C., Kibiryeva, N., Dasouki, M., Knoll, J. H. M., and Butler, M. G. (2006). A 9-year-old male with a duplication of chromosome 3p25.3p26.2: Clinical report and gene expression analysis. *Am. J. Med. Genet. Part A* 140A, 573–579. doi:10.1002/AJMG.A.31132.

Bittel, D. C., Kibiryeva, N., Sell, S. M., Strong, T. V., and Butler, M. G. (2007). Whole genome microarray analysis of gene expression in Prader–Willi syndrome. *Am. J. Med. Genet. Part A* 143A, 430–442. doi:10.1002/ajmg.a.31606.

Blanchet, P., Bebin, M., Bruet, S., Cooper, G. M., Thompson, M. L., Duban-Bedu, B., et al. (2017). MYT1L mutations cause intellectual disability and variable obesity by dysregulating gene expression and development of the neuroendocrine hypothalamus. *PLOS Genet.* 13, e1006957. doi:10.1371/JOURNAL.PGEN.1006957.

Burn, B., Brown, S., and Chang, C. (2011). Regulation of early Xenopus development by the PIAS genes. *Dev. Dyn.* 240, 2120–2126. doi:10.1002/DVDY.22701.

Burns, B., Schmidt, K., Williams, S. R., Kim, S., Girirajan, S., and Elsea, S. H. (2010). Rai1 haploinsufficiency causes reduced Bdnf expression resulting in hyperphagia, obesity and altered fat distribution in mice and humans with no evidence of metabolic syndrome. *Hum. Mol. Genet.* 19, 4026–4042. doi:10.1093/HMG/DDQ317.

Canitano, R., and Pallagrosi, M. (2017). Autism spectrum disorders and schizophrenia spectrum disorders: Excitation/inhibition imbalance and developmental trajectories. *Front. Psychiatry* 8. doi:10.3389/FPSYT.2017.00069.

Capelli, L. P., Krepischi, A. C. V., Gurgel-Giannetti, J., Mendes, M. F. S., Rodrigues, T., Varela, M. C., et al. (2012). Deletion of the RMGA and CHD2 genes in a child with epilepsy and mental deficiency. *Eur. J. Med. Genet.* 55, 132–134. doi:10.1016/J.EJMG.2011.10.004.

Cassidy, S. B., Schwartz, S., Miller, J. L., and Driscoll, D. J. (2012). Prader-Willi syndrome. *Genet. Med.* 14, 10–26. doi:10.1038/gim.0b013e31822bead0.

Cherik, F., Lepage, M., Remerand, G., Francannet, C., Delabaere, A., Salaun, G., et al. (2021). Further refining the critical region of 10q26 microdeletion syndrome: A possible involvement of INSYN2 and NPS in the cognitive phenotype. *Eur. J. Med. Genet.* 64. doi:10.1016/J.EJMG.2021.104287.

Coulson, R. L., Yasui, D. H., Dunaway, K. W., Laufer, B. I., Vogel Ciernia, A., Zhu, Y., et al. (2018). Snord116-dependent diurnal rhythm of DNA methylation in mouse cortex. *Nat. Commun.* 9, 1–11. doi:10.1038/s41467-018-03676-0.

Courage, C., Houge, G., Gallati, S., Schjelderup, J., and Rieubland, C. (2014). 15q26.1 microdeletion encompassing only CHD2 and RGMA in two adults with moderate intellectual disability, epilepsy and truncal obesity. *Eur. J. Med. Genet.* 57, 520–523. doi:10.1016/J.EJMG.2014.06.003.

Coursimault, J., Guerrot, A. M., Morrow, M. M., Schramm, C., Zamora, F. M., Shanmugham, A., et al. (2022). MYT1L-associated neurodevelopmental disorder: description of 40 new cases and literature review of clinical and molecular aspects. *Hum. Genet.* 141, 65–80. doi:10.1007/S00439-021-02383-Z/FIGURES/5.

Courtens, W., Wuyts, W., Rooms, L., Pera, S. B., and Wauters, J. (2006). A subterminal deletion of the long arm of chromosome 10: A clinical report and review. *Am. J. Med. Genet. Part A* 140A, 402–409. doi:10.1002/AJMG.A.31053.

Crespi, B., Read, S., Salminen, I., and Hurd, P. (2018). A genetic locus for paranoia. *Biol. Lett.* 14. doi:10.1098/rsbl.2017.0694.

D’Angelo, C. S., Da Paz, J. A., Kim, C. A., Bertola, D. R., Castro, C. I. E., Varela, M. C., et al. (2006). Prader-Willi-like phenotype: investigation of 1p36 deletion in 41 patients with delayed psychomotor development, hypotonia, obesity and/or hyperphagia, learning disabilities and behavioral problems. *Eur. J. Med. Genet.* 49, 451–460. doi:10.1016/J.EJMG.2006.02.001.

D’Angelo, C. S., Kohl, I., Varela, M. C., De Castro, C. I. E., Kim, C. A., Bertola, D. R., et al. (2010). Extending the phenotype of monosomy 1p36 syndrome and mapping of a critical region for obesity and hyperphagia. *Am. J. Med. Genet. Part A* 152A, 102–110. doi:10.1002/AJMG.A.33160.

Davenport, E. C., Szulc, B. R., Drew, J., Taylor, J., Morgan, T., Higgs, N. F., et al. (2019). Autism and Schizophrenia-Associated CYFIP1 Regulates the Balance of Synaptic Excitation and Inhibition. *Cell Rep.* 26, 2037-2051.e6. doi:10.1016/J.CELREP.2019.01.092.

De Maria, B., Balestrini, S., Mei, D., Melani, F., Pellacani, S., Pisano, T., et al. (2022). Expanding the genetic and phenotypic spectrum of CHD2-related disease: From early neurodevelopmental disorders to adult-onset epilepsy. *Am. J. Med. Genet. Part A* 188, 522–533. doi:10.1002/AJMG.A.62548.

de Smith, A. J., van Haelst, M. M., Ellis, R. J., Holder, S. E., Payne, S. J., Hashim, S. K., et al. (2011). Chromosome 19p13.3 deletion in a patient with macrocephaly, obesity, mental retardation, and behavior problems. *Am. J. Med. Genet. Part A* 155, 1192–1195. doi:10.1002/ajmg.a.33986.

De Taevernier, C., Meunier-Cussac, S., and Madigand, J. (2021). First episode of psychosis in Kleefstra syndrome: a case report. *https://doi.org/10.1080/13554794.2021.1905851* 27, 227–230. doi:10.1080/13554794.2021.1905851.

de Vries, B. B. A., Fryns, J. P., Butler, M. G., Canziani, F., Wesby-van Swaay, E., van Hemel, J. O., et al. (1993). Clinical and molecular studies in fragile X patients with a Prader-Willi-like phenotype. *J. Med. Genet.* 30, 761–766. doi:10.1136/jmg.30.9.761.

Dobson, N. R., Moore, R. T., Tobin, J. E., and Armstrong, R. C. (2012). Leukemia/lymphoma-related factor regulates oligodendrocyte lineage cell differentiation in developing white matter. *Glia* 60, 1378–1390. doi:10.1002/GLIA.22356.

Duplan, S. M., Boucher, F., Alexandrov, L., and Michaud, J. L. (2009). Impact of Sim1 gene dosage on the development of the paraventricular and supraoptic nuclei of the hypothalamus. *Eur. J. Neurosci.* 30, 2239–2249. doi:10.1111/J.1460-9568.2009.07028.X.

Edelman, E. A., Girirajan, S., Finucane, B., Patel, P. I., Lupski, J. R., Smith, A. C. M., et al. (2007). Gender, genotype, and phenotype differences in Smith–Magenis syndrome: a meta-analysis of 105 cases. *Clin. Genet.* 71, 540–550. doi:10.1111/J.1399-0004.2007.00815.X.

Faria, Á. C., Rabbi-Bortolini, E., Rebouças, M. R. G. O., Andréia, A. L. A., Frasson, M. G. T., Atique, R., et al. (2016). Craniosynostosis in 10q26 deletion patients: A consequence of brain underdevelopment or altered suture biology? *Am. J. Med. Genet. Part A* 170, 403–409. doi:10.1002/AJMG.A.37448.

Feighan, S. M., Hughes, M., Maunder, K., Roche, E., and Gallagher, L. (2020). A profile of mental health and behaviour in Prader–Willi syndrome. *J. Intellect. Disabil. Res.* 64, 158–169. doi:10.1111/JIR.12707.

Gilmour, J., and Skuse, D. (1999). A case-comparison study of the characteristics of children with a short stature syndrome induced by stress (Hyperphagic Short Stature) and a consecutive series of unaffected “stressed” children. *J. Child Psychol. Psychiatry Allied Discip.* 40, 969–978. doi:10.1017/S0021963099004230.

Gilmour, J., Skuse, D., and Pembrey, M. (2001). Hyperphagic short stature and Prader-Willi syndrome: A comparison of behavioural phenotypes, genotypes and indices of stress. *Br. J. Psychiatry* 179, 129–137. doi:10.1192/bjp.179.2.129.

Grund, T., and Neumann, I. D. (2019). Brain neuropeptide S: via GPCR activation to a powerful neuromodulator of socio-emotional behaviors. *Cell Tissue Res.* 375, 123–132. doi:10.1007/S00441-018-2902-2/FIGURES/2.

Ingason, A., Kirov, G., Giegling, I., Hansen, T., Isles, A. R., Jakobsen, K. D., et al. (2011). Maternally derived microduplications at 15q11-q13: Implication of imprinted genes in psychotic illness. *Am. J. Psychiatry* 168, 408–417. doi:10.1176/appi.ajp.2010.09111660.

Ioannides, Y., Lokulo-Sodipe, K., Mackay, D. J. G., Davies, J. H., and Temple, I. K. (2014). Temple syndrome: improving the recognition of an underdiagnosed chromosome 14 imprinting disorder: an analysis of 51 published cases. *J. Med. Genet.* 51, 495–501. doi:10.1136/JMEDGENET-2014-102396.

Kagami, M., Nagasaki, K., Kosaki, R., Horikawa, R., Naiki, Y., Saitoh, S., et al. (2017). Temple syndrome: comprehensive molecular and clinical findings in 32 Japanese patients. *Genet. Med.* 19, 1356–1366. doi:10.1038/GIM.2017.53.

Khattabi, L. El, Guimiot, F., Pipiras, E., Andrieux, J., Baumann, C., Bouquillon, S., et al. (2015). Incomplete penetrance and phenotypic variability of 6q16 deletions including SIM1. *Eur. J. Hum. Genet.* 23, 1010–1018. doi:10.1038/ejhg.2014.230.

Kim, M., de la Peña, J. B., Cheong, J. H., and Kim, H. J. (2018a). Neurobiological functions of the period circadian clock 2 gene, per2. *Biomol. Ther.* 26, 358–367. doi:10.4062/BIOMOLTHER.2017.131.

Kim, Y. J., Khoshkhoo, S., Frankowski, J. C., Zhu, B., Abbasi, S., Lee, S., et al. (2018b). Chd2 Is Necessary for Neural Circuit Development and Long-Term Memory. *Neuron* 100, 1180-1193.e6. doi:10.1016/J.NEURON.2018.09.049.

Kleefstra, T., Van Zelst-Stams, W. A., Nillesen, W. M., Cormier-Daire, V., Houge, G., Foulds, N., et al. (2009). Further clinical and molecular delineation of the 9q subtelomeric deletion syndrome supports a major contribution of EHMT1 haploinsufficiency to the core phenotype. *J. Med. Genet.* 46, 598–606. doi:10.1136/JMG.2008.062950.

Kostić, V. S., Stefanova, E., Svetel, M., and Kozić, D. (1998). A variant of the Kleine-Levin syndrome following head trauma. *Behav. Neurol.* 11, 105–108. doi:10.1155/1998/413989.

Kotler, J., Balko, K., Berall, G., and Haig, D. (2016). Nutritional Phases in Prader-Willi Syndrome: Evolutionary and Clinical Interpretations. *J. Evol. Med.* 4, 1–7. doi:10.4303/jem/235968.

Le, T. N., Williams, S. R., Alaimo, J. T., and Elsea, S. H. (2019). Genotype and phenotype correlation in 103 individuals with 2q37 deletion syndrome reveals incomplete penetrance and supports HDAC4 as the primary genetic contributor. *Am. J. Med. Genet. Part A* 179, 782–791. doi:10.1002/AJMG.A.61089.

Lee, V., Sarkar, J., and Maguire, J. (2014). Loss of Gabrd in CRH neurons blunts the corticosterone response to stress and diminishes stress-related behaviors. *Psychoneuroendocrinology* 41, 75–88. doi:10.1016/J.PSYNEUEN.2013.12.011.

Leroy, C., Landais, E., Briault, S., David, A., Tassy, O., Gruchy, N., et al. (2013). The 2q37-deletion syndrome: an update of the clinical spectrum including overweight, brachydactyly and behavioural features in 14 new patients. *Eur. J. Hum. Genet.* 21, 602–612. doi:10.1038/ejhg.2012.230.

Lin, S., Zhou, Y., Fang, Q., Wu, J., Zhang, Z., Ji, Y., et al. (2016). Chromosome 10q26 deletion syndrome: Two new cases and a review of the literature. *Mol. Med. Rep.* 14, 5134–5140. doi:10.3892/MMR.2016.5864/HTML.

Lopez, S. J., Segal, D. J., and LaSalle, J. M. (2019). UBE3A: An E3 ubiquitin ligase with genome-wide impact in neurodevelopmental disease. *Front. Mol. Neurosci.* 11, 1–8. doi:10.3389/fnmol.2018.00476.

MacDonald, K., and MacDonald, T. M. (2010). The peptide that binds: A systematic review of Oxytocin and its prosocial effects in humans. *Harv. Rev. Psychiatry* 18, 1–21. doi:10.3109/10673220903523615.

Maguire, J., and Mody, I. (2008). GABAAR Plasticity during Pregnancy: Relevance to Postpartum Depression. *Neuron* 59, 207–213. doi:10.1016/J.NEURON.2008.06.019.

Marbach, F., Elgizouli, M., Rech, M., Beygo, J., Erger, F., Velmans, C., et al. (2020). The adult phenotype of Schaaf-Yang syndrome. *Orphanet J. Rare Dis.* 15. doi:10.1186/S13023-020-01557-8.

McCarthy, J., Lupo, P. J., Kovar, E., Rech, M., Bostwick, B., Scott, D., et al. (2018). Schaaf-Yang syndrome overview: Report of 78 individuals. *Am. J. Med. Genet. A* 176, 2564–2574. doi:10.1002/ajmg.a.40650.

Mercer, R. E., Michaelson, S. D., Chee, M. J. S., Atallah, T. A., Wevrick, R., and Colmers, W. F. (2013). Magel2 Is Required for Leptin-Mediated Depolarization of POMC Neurons in the Hypothalamic Arcuate Nucleus in Mice. *PLoS Genet.* 9. doi:10.1371/journal.pgen.1003207.

Mielcarek, M., Zielonka, D., Carnemolla, A., Marcinkowski, J. T., and Guidez, F. (2015). HDAC4 as a potential therapeutic target in neurodegenerative diseases: A summary of recent achievements. *Front. Cell. Neurosci.* 9, 42. doi:10.3389/FNCEL.2015.00042/BIBTEX.

Miller, M. F., Cohen, E. D., Baggs, J. E., Hogenesch, J. B., and Morrisey, E. E. (2013). High Throughput Genomic Screen Identifies Multiple Factors That Promote Cooperative Wnt Signaling. *PLoS One* 8, e55782. doi:10.1371/JOURNAL.PONE.0055782.

Nag, H. E., Nordgren, A., Anderlid, B. M., and Nærland, T. (2018). Reversed gender ratio of autism spectrum disorder in Smith-Magenis syndrome. *Mol. Autism* 9. doi:10.1186/s13229-017-0184-2.

Natera-de Benito, D., García-Pérez, M. A., Martínez-Granero, M. Á., and Izquierdo-López, L. (2014). A patient with a duplication of chromosome 3p (p24.1p26.2): A comparison with other partial 3p trisomies. *Am. J. Med. Genet. Part A* 164, 548–550. doi:10.1002/ajmg.a.36164.

Nevado, J., Rosenfeld, J. A., Mena, R., Palomares-Bralo, M., Vallespín, E., Mori, M. Á., et al. (2015). PIAS4 is associated with macro/microcephaly in the novel interstitial 19p13.3 microdeletion/microduplication syndrome. *Eur. J. Hum. Genet.* 23, 1615–1626. doi:10.1038/EJHG.2015.51.

Nicholls, R. D., and Knepper, J. L. (2001). Genome Organization, Function, and Imprinting in Prader-Willi and Angelman Syndrome. *Annu. Rev. Genomics Hum. Genet.* 2, 153–175. doi:10.1146/annurev.genom.2.1.153.

Noor, A., Dupuis, L., Mittal, K., Lionel, A. C., Marshall, C. R., Scherer, S. W., et al. (2015). 15q11.2 Duplication Encompassing Only the UBE3A Gene Is Associated with Developmental Delay and Neuropsychiatric Phenotypes. *Hum. Mutat.* 36, 689–693. doi:10.1002/humu.22800.

Nowicki, S. T., Tassone, F., Ono, M. Y., Ferranti, J., Croquette, M. F., Goodlin-Jones, B., et al. (2007). The Prader-Willi phenotype of fragile X syndrome. *J. Dev. Behav. Pediatr.* 28, 133–138. doi:10.1097/01.DBP.0000267563.18952.C9.

Ohishi, A., Masunaga, Y., Iijima, S., Yamoto, K., Kato, F., Fukami, M., et al. (2020). De novo ZBTB7A variant in a patient with macrocephaly, intellectual disability, and sleep apnea: implications for the phenotypic development in 19p13.3 microdeletions. *J. Hum. Genet.* 65, 181–186. doi:10.1038/S10038-019-0690-5.

Persson-Augner, D., Lee, Y. W., Tovar, S., Dieguez, C., and Meister, B. (2014). Delta-Like 1 Homologue (DLK1) protein in neurons of the arcuate nucleus that control weight homeostasis and effect of fasting on hypothalamic DLK1 mRNA. *Neuroendocrinology* 100, 209–220. doi:10.1159/000369069.

Rao, N. R., Abad, C., Perez, I. C., Srivastava, A. K., Young, J. I., and Walz, K. (2017). Rai1 haploinsufficiency is associated with social abnormalities in mice. *Biology (Basel).* 6. doi:10.3390/BIOLOGY6020025.

Rosenfeld, J. A., Crolla, J. A., Tomkins, S., Bader, P., Morrow, B., Gorski, J., et al. (2010). Refinement of causative genes in monosomy 1p36 through clinical and molecular cytogenetic characterization of small interstitial deletions. *Am. J. Med. Genet. Part A* 152, 1951–1959. doi:10.1002/AJMG.A.33516.

Salminen, I. I., Crespi, B. J., and Mokkonen, M. (2019). Baby food and bedtime: Evidence for opposite phenotypes from different genetic and epigenetic alterations in Prader-Willi and Angelman syndromes. *SAGE Open Med.* 7, 205031211882358. doi:10.1177/2050312118823585.

Schrander‐Stumpel, C., Gerver, W. ‐J, Engelen, J., Mulder, H., and Fryns, J. ‐P (1994). Prader‐Willi‐like phenotype in fragile X syndrome. *Clin. Genet.* 45, 175–180. doi:10.1111/j.1399-0004.1994.tb04018.x.

Soni, S., Whittington, J., Holland, A. J., Webb, T., Maina, E., Boer, H., et al. (2007). The course and outcome of psychiatric illness in people with Prader-Willi syndrome: Implications for management and treatment. *J. Intellect. Disabil. Res.* 51, 32–42. doi:10.1111/j.1365-2788.2006.00895.x.

Stelzer, Y., Sagi, I., Yanuka, O., Eiges, R., and Benvenisty, N. (2014). The noncoding RNA IPW regulates the imprinted DLK1-DIO3 locus in an induced pluripotent stem cell model of Prader-Willi syndrome. *Nat. Genet.* 46, 551–557. doi:10.1038/NG.2968.

Stevens, S. J. C., van Ravenswaaij-Arts, C. M. A., Janssen, J. W. H., Klein Wassink-Ruiter, J. S., van Essen, A. J., Dijkhuizen, T., et al. (2011). MYT1L is a candidate gene for intellectual disability in patients with 2p25.3 (2pter) deletions. *Am. J. Med. Genet. Part A* 155, 2739–2745. doi:10.1002/AJMG.A.34274.

Tenorio, J., Nevado, J., González-Meneses, A., Arias, P., Dapía, I., Venegas-Vega, C. A., et al. (2020). Further definition of the proximal 19p13.3 microdeletion/microduplication syndrome and implication of PIAS4 as the major contributor. *Clin. Genet.* 97, 467–476. doi:10.1111/CGE.13689/V1/REVIEW2.

Thygesen, J. H., Wolfe, K., McQuillin, A., Viñas-Jornet, M., Baena, N., Brison, N., et al. (2018). Neurodevelopmental risk copy number variants in adults with intellectual disabilities and comorbid psychiatric disorders. *Br. J. Psychiatry* 212, 287–294. doi:10.1192/BJP.2017.65.

Varela, M. C., Simões-Sato, A. Y., Kim, C. A., Bertola, D. R., De Castro, C. I. E., and Koiffmann, C. P. (2006). A new case of interstitial 6q16.2 deletion in a patient with Prader–Willi-like phenotype and investigation of SIM1 gene deletion in 87 patients with syndromic obesity. *Eur. J. Med. Genet.* 49, 298–305. doi:10.1016/J.EJMG.2005.12.002.

Verhoeven, W. M. A., Egger, J. I. M., Knegt, A. C., Zuydam, J., and Kleefstra, T. (2016). Absence epilepsy and the CHD2 gene: An adolescent male with moderate intellectual disability, short-lasting psychoses, and an interstitial deletion in 15q26.1–q26.2. *Neuropsychiatr. Dis. Treat.* 12, 1135–1139. doi:10.2147/NDT.S102272.

Verhoeven, W. M. A., Egger, J. I. M., Vermeulen, K., van de Warrenburg, B. P. C., and Kleefstra, T. (2011). Kleefstra syndrome in three adult patients: Further delineation of the behavioral and neurological phenotype shows aspects of a neurodegenerative course. *Am. J. Med. Genet. Part A* 155, 2409–2415. doi:10.1002/AJMG.A.34186.

Villavicencio-Lorini, P., Klopocki, E., Trimborn, M., Koll, R., Mundlos, S., and Horn, D. (2012). Phenotypic variant of Brachydactyly-mental retardation syndrome in a family with an inherited interstitial 2q37.3 microdeletion including HDAC4. *Eur. J. Hum. Genet. 2013 217* 21, 743–748. doi:10.1038/ejhg.2012.240.

Wattchow, N., Lee, H. E., and Brock, P. (2015). Psychosocial short stature with psychosis: A case report. *Australas. Psychiatry* 23, 63–65. doi:10.1177/1039856214563844.

Yatsenko, S. A., Kruer, M. C., Bader, P. I., Corzo, D., Schuette, J., Keegan, C. E., et al. (2009). Identification of critical regions for clinical features of distal 10q deletion syndrome. *Clin. Genet.* 76, 54–62. doi:10.1111/J.1399-0004.2008.01115.X.
