# Supplementary material for: Do the diverse phenotypes of Prader-Willi syndrome reflect extremes of covariation in typical populations?
Source: Front Genet. 2022 Nov 24;13:1041943. doi: 10.3389/fgene.2022.1041943 (PMC9731222; doi:10.3389/fgene.2022.1041943)
Supplement: Supplementary file 2 [file Table2.DOCX]

| Gene | Neural mechanisms identified | Behavioral alterations shown deletions |
| --- | --- | --- |
| Dio3 | Alterations in oxytocin signaling (1) | Reduced pup retrieval and interactions between mother and offspring, anxiety and depression –like behaviors (1,2) |
| Peg3 | Peg3 may take part in regulating the expression Oxtr (3) | Reduced exploration and reduced matermal care (2,4,5), Peg3KO pups show reduced ultrasonic vocalizations during separation from their mothers (6) |
| Dlk1 | Orexin-expressing neurons (7), may also take part in regulation of neural development via NOTCH pathway (8), may interact with the PWS locus during transcription (9) | Anxiety –like behaviors (7), and metabolic growth restrictions (10) Altered DLK1 function has beem associated with psychotic disorders (11) |
| Rtl1 | Expressed in hypothalamus, other brain regions (12) | Impaired suckling, increase in anxiety and depression –like behaviors, avoidance towards social novelty (12). |
| GNASxl | Alternatively spliced transcript active in brain, particularly pituitary gland and hypothalamus (13), may be related to mTOR1-S6K pathway function in the hypothalamus (14) | Impaired suckling, failure to thrive (13,14) |
| Nnat | Interacts with leptin in hypothalamus (15) | Postnatal growth restriction and obesity in adulthood (16), polymorphisms also associated with obesity in humans (15). |
| Zdbf2 | Localized expression in HPA axis, appears to NPY expression in arcuate nucleus (17), preferentially expressed from the paternal allele in the brain, regulated by a maternal DMR during embryogenesis (18) | Reduced suckling, growth restriction if deleted, increased biallelic dosage associated with a small increase in weight (17). |
| Grb10 | Expressed from maternal allele in body, paternal allele in the brain (19) | Lack of expression associated with increased social dominance (19). Grb10KO pups show increased weight and suckling in offspring but only when fostered by WT mothers, expression in maternal genotype reduces fat mass among offspring (20) |
| Peg13 | Active in hypothalamus (21), altered expression of estrogen receptors, other neurotransmitter receptors (22) | Deficiency in pup retrieval, increased anxiety, preference for social interactions with same (rather than opposite) sex, may also indicate altered sexual preference (22) |

(1) (Stohn et al., 2018), (2) (Creeth et al., 2019) (3) (Frey et al., 2018) (4) (Champagne et al., 2009) (5) (Chiavegatto et al., 2012) (6) (McNamara et al., 2018) (7) (Harris et al., 2020), (8) (Surmacz et al., 2012), (9) (Stelzer et al., 2014), (10) (Traustadóttir et al., 2019), (11) (Hoseth et al.) (12) (Chou et al., 2022) (13) (Plagge et al., 2004) (14) (Geneviève et al., 2005) (15) (Vrang et al., 2010) (16) (Millership et al., 2018) (17) (Glaser et al., 2022) (18) (Greenberg et al., 2016) (19) (Garfield et al., 2011) (20) (Cowley et al., 2014) (21) (Davies et al., 2004) (22) (Keshavarz and Tautz, 2021)

Supplementary Table 2. Paternally expressed imprinted genes active in hypothalamus showing phenotypes relevant to affection and maternal investment in mouse model studies and neurogenetic disorders.

Champagne, F. A., Curley, J. P., Swaney, W. T., Hasen, N. S., and Keverne, E. B. (2009). Paternal Influence on Female Behavior: The Role of Peg3 in Exploration, Olfaction, and Neuroendocrine Regulation of Maternal Behavior of Female Mice. *Behav. Neurosci.* 123, 469–480. doi:10.1037/A0015060.

Chiavegatto, S., Sauce, B., Ambar, G., Cheverud, J. M., and Peripato, A. C. (2012). Hypothalamic expression of Peg3 gene is associated with maternal care differences between SM/J and LG/J mouse strains. *Brain Behav.* 2, 365–376. doi:10.1002/BRB3.58.

Chou, M.-Y., Hu, M.-C., Chen, P.-Y., Hsu, C.-L., Lin, T.-Y., Tan, M.-J., et al. (2022). RTL1/PEG11 imprinted in human and mouse brain mediates anxiety-like and social behaviors and regulates neuronal excitability in the locus coeruleus. *Hum. Mol. Genet.* doi:10.1093/HMG/DDAC110.

Cowley, M., Garfield, A. S., Madon-Simon, M., Charalambous, M., Clarkson, R. W., Smalley, M. J., et al. (2014). Developmental Programming Mediated by Complementary Roles of Imprinted Grb10 in Mother and Pup. *PLOS Biol.* 12, e1001799. doi:10.1371/JOURNAL.PBIO.1001799.

Creeth, H. D. J., McNamara, G. I., Isles, A. R., and John, R. M. (2019). Imprinted genes influencing the quality of maternal care. *Front. Neuroendocrinol.* 53, 100732. doi:10.1016/J.YFRNE.2018.12.003.

Davies, W., Smith, R. J., Kelsey, G., and Wilkinson, L. S. (2004). Expression patterns of the novel imprinted genes Nap1l5 and Peg13 and their non-imprinted host genes in the adult mouse brain. *Gene Expr. Patterns* 4, 741–747. doi:10.1016/J.MODGEP.2004.03.008.

Frey, W. D., Sharma, K., Cain, T. L., Nishimori, K., Teruyama, R., and Kim, J. (2018). Oxytocin receptor is regulated by Peg3. *PLoS One* 13, e0202476. doi:10.1371/journal.pone.0202476.

Garfield, A. S., Cowley, M., Smith, F. M., Moorwood, K., Stewart-Cox, J. E., Gilroy, K., et al. (2011). Distinct physiological and behavioural functions for parental alleles of imprinted Grb10. *Nature* 469, 534–540. doi:10.1038/NATURE09651.

Geneviève, D., Sanlaville, D., Faivre, L., Kottler, M. L., Jambou, M., Gosset, P., et al. (2005). Paternal deletion of the GNAS imprinted locus (including Gnasxl) in two girls presenting with severe pre- and post-natal growth retardation and intractable feeding difficulties. *Eur. J. Hum. Genet.* 13, 1033–1039. doi:10.1038/SJ.EJHG.5201448.

Glaser, J., Iranzo, J., Borensztein, M., Marinucci, M., Gualtieri, A., Jouhanneau, C., et al. (2022). The imprinted Zdbf2 gene finely tunes control of feeding and growth in neonates. *Elife* 11. doi:10.7554/ELIFE.65641.

Greenberg, M. V. C., Glaser, J., Borsos, M., Marjou, F. El, Walter, M., Teissandier, A., et al. (2016). Transient transcription in the early embryo sets an epigenetic state that programs postnatal growth. *Nat. Genet. 2016 491* 49, 110–118. doi:10.1038/ng.3718.

Harris, T., Bugescu, R., Kelly, J., Makela, A., Sotzen, M., Sisk, C., et al. (2020). Dlk1 expressed in mouse orexin neurons modulates anxio-depressive behavior but not energy balance. *Brain Sci.* 10, 1–18. doi:10.3390/BRAINSCI10120975.

Hoseth, E. Z., Krull, F., Dieset, I., Mørch, R. H., Hope, S., Gardsjord, E. S., et al. Attenuated Notch signaling in schizophrenia and bipolar disorder. doi:10.1038/s41598-018-23703-w.

Keshavarz, M., and Tautz, D. (2021). The imprinted lncRNA Peg13 regulates sexual preference and the sex-specific brain transcriptome in mice. *Proc. Natl. Acad. Sci. U. S. A.* 118. doi:10.1073/PNAS.2022172118.

McNamara, G. I., Creeth, H. D. J., Harrison, D. J., Tansey, K. E., Andrews, R. M., Isles, A. R., et al. (2018). Loss of offspring Peg3 reduces neonatal ultrasonic vocalizations and increases maternal anxiety in wild-type mothers. *Hum. Mol. Genet.* 27, 440–450. doi:10.1093/HMG/DDX412.

Millership, S. J., Tunster, S. J., Van de Pette, M., Choudhury, A. I., Irvine, E. E., Christian, M., et al. (2018). Neuronatin deletion causes postnatal growth restriction and adult obesity in 129S2/Sv mice. *Mol. Metab.* 18, 97–106. doi:10.1016/J.MOLMET.2018.09.001.

Plagge, A., Gordon, E., Dean, W., Boiani, R., Cinti, S., Peters, J., et al. (2004). The imprinted signaling protein XLαs is required for postnatal adaptation to feeding. *Nat. Genet.* 36, 818–826. doi:10.1038/NG1397.

Stelzer, Y., Sagi, I., Yanuka, O., Eiges, R., and Benvenisty, N. (2014). The noncoding RNA IPW regulates the imprinted DLK1-DIO3 locus in an induced pluripotent stem cell model of Prader-Willi syndrome. *Nat. Genet.* 46, 551–557. doi:10.1038/NG.2968.

Stohn, J. P., Martinez, M. E., Zafer, M., López-Espíndola, D., Keyes, L. M., and Hernandez, A. (2018). Increased aggression and lack of maternal behavior in Dio3-deficient mice are associated with abnormalities in oxytocin and vasopressin systems. *Genes, Brain Behav.* 17, 23–35. doi:10.1111/GBB.12400.

Surmacz, B., Noisa, P., Risner-Janiczek, J. R., Hui, K., Ungless, M., Cui, W., et al. (2012). DLK1 Promotes Neurogenesis of Human and Mouse Pluripotent Stem Cell-Derived Neural Progenitors Via Modulating Notch and BMP Signalling. *Stem Cell Rev. Reports* 8, 459–471. doi:10.1007/s12015-011-9298-7.

Traustadóttir, G. Á., Lagoni, L. V., Ankerstjerne, L. B. S., Bisgaard, H. C., Jensen, C. H., and Andersen, D. C. (2019). The imprinted gene Delta like non-canonical Notch ligand 1 (Dlk1)is conserved in mammals, and serves a growth modulatory role during tissue development and regeneration through Notch dependent and independent mechanisms. *Cytokine Growth Factor Rev.* 46, 17–27. doi:10.1016/j.cytogfr.2019.03.006.

Vrang, N., Meyre, D., Froguel, P., Jelsing, J., Tang-Christensen, M., Vatin, V., et al. (2010). The imprinted gene neuronatin is regulated by metabolic status and associated with obesity. *Obesity* 18, 1289–1296. doi:10.1038/OBY.2009.361.
